# Incorporation of fresh leaves of wormwood (*Artemisia herba alba*) and/or rosemary (*Rosmarinus officinalis*) in the diet of rams: Effect on testicular function, sexual behavior, and blood parameters

**DOI:** 10.1002/fsn3.3293

**Published:** 2023-03-28

**Authors:** Samia Khnissi, Giovanni Bomboi, Ikram Khémiri, Imen Ben Salem, Maria Dattena, Salma Sai, Souha Ben Mustapha, Andrea Cabiddu, Narjess Lassoued

**Affiliations:** ^1^ Laboratory of Animal and Forage Production, National Institute of Agronomic Research of Tunisia (INRAT) University of Carthage Tunis Tunisia; ^2^ Dipartimento di Medicina Veterinaria University of Sassari Sassari Italy; ^3^ Unit of Physiology of Regulatory Systems and Adaptations (UR17ES10), Department of Biology, Faculty of Sciences of Tunis University of Tunis El Manar Tunis Tunisia; ^4^ Département des Productions Animales, Service de Zootechnie et Economie Agricole Ecole Nationale de Médecine Vétérinaire Sidi Thabet Tunisie; ^5^ Department of Animal Science Agricultural Research Agency of Sardinia Olmedo Italy

**Keywords:** *Artemisia herba alba*, behavior, ram, *Rosmarinus officinalis*, sexual, sperm

## Abstract

This study aimed to investigate the effect of wormwood and rosemary supplementation on some reproductive traits of Barbarine rams. The experiment lasted 2 months. Twenty‐four adult rams were divided into four groups (*n* = 6) balanced for the weight (53.3 ± 1.2 kg body weight [BW] ± SD). All rams received 1200 g of straw and 600 g of barley. Control rams (C) without aromatic medicinal plant (AMP), while experimental rams received 20 g of fresh rosemary leaves (R), 20 g of fresh wormwood leaves (A), and 10 g of fresh rosemary leaves plus 10 g of fresh wormwood leaves (RA). The results revealed that the live weight of all rams increased (*p* < .05) in the RA group compared to the C, A, and R groups. Scrotal circumference increased in the R rams when compared to the controls rams (*p* < .05). For sperm parameters we showed that the A rams had higher sperm concentrations (*p* < .05). But, the sperm volume decreased in the R rams (*p >* .05). However, when the rams received rosemary plus wormwood, their sperm volume increased (*p* > .05). The sperm mass motility was higher for the A, R and AR rams in comparison to the C rams (*p* = .05). On the other hand, biochemical analysis of the seminal fluid showed no effect of diets on calcium and total proteins concentration. But the measurement of glucose and seminal insulin showed a decrease (*p* < .05) in these two biochemical markers in group A rams and a decrease (*p* < .05) in insulin without modification of the glucose concentration in R rams. Blood glucose and insulin decreased in the animals on AMP diet compared to the other groups (*p* < .05) while aspartate aminotransferase (AST) increased (*p* < .05). Rosemary leaves (R and RA groups) increased (*p* < .05) plasma cortisol compared to the other groups. It can be concluded that the addition of *Rosmarinus officinalis* and/or *Artemisia herba alba* in ram diet can have a positive effect on the reproductive function by increasing the concentration and motility of sperm, plasma testosterone, and sexual behavior.

## Graphical abstract

This study is part of the research that supports natural and ecological methods to improve reproductive performances and livestock productivity. In this paper we show that the consumption of small quantities of AMPs (*Artemisia herba alba* and/or *Rosmarinus officinalis*), which are naturally abundant in the Mediterranean rangeland can present an opportunity to improve the productivity of ecological sheep farming by improving the performance and reproductive health of rams.

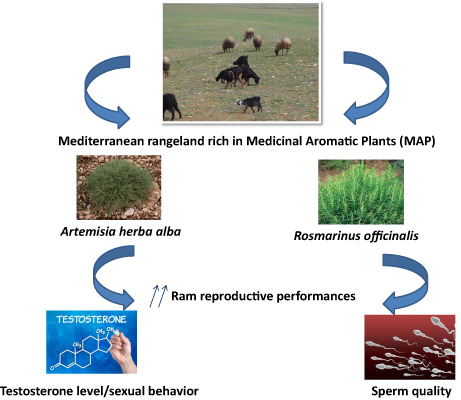

## 1 INTRODUCTION

Reproductive performance is an important parameter affecting flock profitability (Pardos et al., 2008), in which the reproductive capacity of the rams plays an important role. In fact, 50% of the reproductive potential of a flock is provided by the ram (MacLaren, 1988). Food supplementation with plants rich in active molecules can present a natural and ecological opportunity to improve the reproductive health of farm animals.

Nowadays, medicinal plants are widely used all over the world as an alternative to pharmaceutical drugs. Medicinal plants and their derivatives, rich in active molecules, are widely studied for their anti‐inflammatory, antibacterial, antifungal, antiulcer, wound healing anti‐cancer and sedative activities (Ben et al., 2018; Ghannadi et al., 2004; Inatani et al., 1983; Juhás et al., 2009; Khémiri et al., 2020; Khémiri & Bitri, 2019; Maiza et al., 2020; Mirdeilami et al., 2011; Ramezani et al., 2008; Rebai et al., 2021). Many studies have shown the effect of some medicinal plants on male reproductive performance such as live weight, testicular growth, sperm production, gamete quality, and sexual behavior (Blache et al., 2003; Martin et al., 1999; Martin & Walkden‐Brown, 1995). *Artemisia herba alba* (Asteraceae) and *Rosmarinus officinalis* (Lamiaceae) are aromatic and medicinal shrubs abundant in Mediterranean rangeland including Tunisia. Some studies have shown the pharmacological interest of these plants as antioxidant, antimicrobial, antiparasitic, and rumen functions (Cobellis et al., 2015; Ekhlil et al., 2016; Shaalan, 2015). However, there is little research that has focused on their effect on reproductive performance, mainly in male reproduction. In female performances (Laadraoui et al., 2018) showed the administration of 80 and 150 mg/kg/day of methanol extract of *Artemisia herba alba* during the entire period of gestation decreases the fertility of Swiss mice. However, (Selmi et al., 2016) showed that essential oil has a positive effect on male mice reproduction. Regarding *Rosmarinus officinalis* (Elmi et al., 2017) showed a total spermicidal effect on human spermatozoa at 0.8 mg mL^−1^ compared to *Artemisia herba alba*. However, (Touazi et al., 2018) showed that small concentrations of *Rosmarinus officinalis* essential oil increase the fertility of cocks' spermatozoa in vitro and after artificial insemination. Moreover, *Rosmarinus officinalis* affects some hormones and cellules on the testes. It decreased the testosterone level in rats. However, it increased the number of spermatogonia, spermatocyte, Leydig cell and spermatid (Heidari‐Vala et al., 2013).

To our knowledge, this is the first in vivo study that has looked into the possible effects of *Artemisia herba alba* and *Rosmarinus officinalis* fresh leaves supplementation on semen quality, biochemical profiles of semen, testicular size, sexual behavior, serum testosterone, and some blood parameters.

## 2 MATERIALS AND METHODS

### 2.1 Location and animals

The trial was carried out in the sheep research farm of the National Institute of Agricultural Research in Tunisia (INRAT). Twenty‐four adult rams of the Barbarine breed (mean live weight 53.3 ± 1.2 kg at the start of the experiment) were selected from the farm flock on the basis of their ability to ejaculate in an artificial vagina. The animals were reared under similar conditions and were continuously exposed to natural photoperiod. Prior to the experiment, the animals were treated against internal parasites using Oxfendazol (Medivet, Soliman, Tunisia) at a conventional dose of 5 mg/kg body weight (BW) and vaccinated against enterotoxaemia.

### 2.2 Experimental protocol

The experiment lasted 2 months (March 15‐May 15). The animals were divided into four groups balanced for live weight and were placed in individual boxes (2m^2^ each) inside a barn, well ventilated with wide windows on all sides so that animals had continuous exposure to natural daylight. Each of the 24 rams was fed on a daily basis a diet composed of a mixture of 1200 g (1044 g dry matter (DM)) of wheat straw and 600 g (552 g DM) of barley grains, which was offered into two equal meals (at 08:00 and 19:00 h). Rams in treatment C (control) received a diet without aromatic and medicinal plants (AMP), while experimental rams received 20 g (8 g DM) of fresh Rosemary (R), 20 g (5.8 g DM) of fresh Artemisia (A) and rams received 10 g (4 g DM) of fresh *Rosemary officinalis* plus 10 g (2.9 g DM) of fresh *Artemisia herba alba* (RA) (Table 1). The experiment lasted 60 days. All animals were subjected to an adaptation period that lasted 15 days.

**TABLE 1:** Food distribution according to group's diets in gram of dry matter.

Food	C	R	A	RA
Straw (g DM)	1044	1044	1044	1044
Barley (g DM)	552	552	552	552
Rosemary (g DM)	0	8	0	4
Wormwood (g DM)	0	0	5.8	2.9
Total diet (g DM)	1596	1604	1601.8	1605.9

Abbreviation: g DM, grams of dry matter.

## 3 SAMPLES AND MEASUREMENTS

### 3.1 Feed and water intake

Feed and water intake were daily assessed for each individual ram by measuring the quantity of feed refusals.

### 3.2 Body weight and testicular circumference

Animal live weight was measured before the distribution of the morning meal. Testicular circumference was measured using a caliber on the same occasions.

### 3.3 Blood parameters

Blood samples were taken every 15 days in the morning before feeding and watering. Blood was withdrawn from the jugular vein using heparinized 10 mL vacuum tubes, centrifuged for 15 min at 3000 ***g***, and the recovered plasma was aliquoted and then stored at −20°C before it was assayed, in the Department of Veterinary Medicine at the University of Sassari, Italy, for biochemical analyses:

- Glucose, total proteins, urea, non‐esterified fatty acid (NEFA), alanine aminotransferase (ALT), and aspartate aminotransferase (AST) was determined using commercial kits (ALCYON (Italy)‐Mindray) on electrolyte analyzer (Model BS200 of Mindray Medical Company (Milan, Italy)).
- Plasma testosterone in ovine was determined using DRG Testosterone ELISA EIA‐1559. DRG Instruments GmbH, Germany (The DRG Testosterone ELISA is an enzyme immunoassay for the quantitative in vitro diagnostic measurement of testosterone in serum and plasma).
- Plasma cortisol was determined using Demeditec Cortisol ELISA DEH3388 (Demeditec Diagnostics GmbH Germany, The DEMEDITEC Cortisol ELISA is a competitive immunoassay for the quantitative in vitro diagnostic measurement of cortisol in serum and plasma, EDTA).
- Plasma insulin was measured by Insulin Mercodia AB. Uppsala (Sweden Mercodia Ovine Insulin ELISA, 10‐1202‐01, provides a method for the quantitative determination of insulin in ovine serum and plasma).

### 3.4 Semen parameters

Collection of semen was done using rams that were put individually in the collection room in presence of a teaser female that was previously inducted into estrus. Estrus was induced by inserting a progestogen‐impregnated vaginal sponge for 6–7 days followed by daily injections of 250 μg of estradiol benzoate for three consecutive days.

Semen traits were assessed every 15 days at the same day of blood sampling. One ejaculate per ram was collected to assess for sperm volume, motility, concentration, and for some biochemical parameters of seminal plasma.

Ejaculates are recovered in glass tubes (4 mL) graduated to the nearest 0.1 mm. This allowed direct determination of the ejaculate volume without considering the frothy part on the top. Concentration (the number of spermatozoa/mL) was determined using a spectrophotometer calibrated to measure sheep sperm concentration at 550 nm (Accucell R; IMV, Paris, France). Four μl of fresh semen were diluted in 3996 μL of physiological saline solution. Mass activity (wave motion or motility score) in undiluted semen was assessed by examining a drop of semen under a warm stage phase contrast microscope at 40× magnification (score, 0–5).

Each ejaculate was then centrifuged for 15 min at 1500 ***g*** and recovered seminal plasma was aliquoted and stored at −80°C before it was assayed by an automatic chemistry chemiluminescence analyzer working DIMENSION RXL, Siemens Company (Milan, Italy) for glucose (Glucose kit ALCYON (Italy)‐Mindray, U.V. (God‐Pod Method)), total proteins (adaptation of the Biuret Method), insulin (Insulin Mercodia AB. Uppsala. Sweden, Mercodia Ovine Insulin ELISA, 10‐1202‐01) and calcium (adaptation of the calcium‐ortho‐cresolphthalein method).

### 3.5 Sexual behavior and testosterone level

Sexual behavior was assessed with rams ordered in a random sequence and exposed to two ewes in estrus for a 15‐min period in a test pen of 6 m^2^. The test facility was constructed to eliminate outside distractions and to prohibit the rams being tested from seeing any sheep other than the teaser ewes. Libido was evaluated by observing mating sexual approaches included vulva sniffing, flehmen, lateral approaches, and mount attempts without ejaculation. Latency to first reaction (time separating introduction of the rams in the pen and observation of the first of any of the behavior traits) and total activity time was also recorded (Kilgour & Whale, 1980).

### 3.6 Statistical analysis

The data obtained are presented as means ± SD. For all traits that were measured for AMP rams and the corresponding measures for C animals, measurements/ram was averaged, and statistical analysis was based on these means. The data statistical analysis was performed according to the MIXED models' procedure (SAS version 9.1; SAS Inst., Cary, NC, USA, 2005). Sources of variation included treatment and measurement time. The random variable was rams' diets. Levels of statistical significance were set at *p* < .05.

## 4 RESULTS

### 4.1 Diet composition, daily feed intake

Analysis of the chemical composition of the different components of the diet showed that the highest percentage of crude protein was recorded in *Artemisia herba alba* and that the leaves of *Rosmarinus officinalis* were richer in water compared to the leaves of *Artemisia herba alba* (Table 2).

**TABLE 2:** Chemical composition of the different foods of the diet (*n* = 8).

	Straw	Barley	*Rosmarinus officinalis*	*Artemisia herba alba*
DM (%)	87	92	40	29
OM (%)	93.26	97.43	93.1	90.36
CP (%)	2.7	10.6	7.83	21.03
NDF (%)	80.17	31.73	40.3	44.23
ADF (%)	53.27	11.17	37.9	23.13
ADL (%)	10.13	1.7	23.13	26.83

Abbreviations: ADF, Acid Detergent Fiber; ADL, acid detergent lignin; CP, crude protein; DM, dry matter; NDF, neutral detergent fiber; OM, organic matter.

Daily control of feedstuff intake showed that there is no difference between the different groups. In fact, the incorporation of the fresh leaves of *Artemisia herba alba* and/or *Rosmarinus officinalis* did not affect the feeding behavior of the animals (Table 3).

**TABLE 3:** Effect of *Artemisia herba alba* and *Rosmarinus officinalis* leaves *supplementation* on *ram semen* quantity, quality, and some seminal fluid biochemical markers; means ± SD.

Parameters	Treatment				Effect, *p*		Treatment × time
	C	R	A	R‐A	Treatment	Time	
Volume (mL)	0.72 ± 0.05^a^	0.55 ± 0.06^a^	0.60 ± 0.05^a^	0.74 ± 0.06^a^	NS	NS	NS
Concentration (×10^6^ spz/mL)	4835 ± 257^b^	4954 ± 268^b^	5134 ± 248^a^	4995 ± 279^a^	<.05	<.01	NS
Massal motility (0–5)	3.19 ± 0.15^a^	3.9 ± 0.16^b^	4.1 ± 0.11^b^	4.02 ± 0.15^b^	<.05	NS	NS
Semen protein (g/dL)	1.33 ± 0.3^a^	1.45 ± 0.3^a^	1.29 ± 0.2^a^	1.58 ± 0.4^a^	NS	NS	NS
Semen glucose (mg/dL)	4.81 ± 1.7^a^	6.82 ± 5.5^b^	1.58 ± 1.7^c^	4.52 ± 3.9^a^	<.01	NS	<.05
Semen insulin (μg/dL)	0.45 ± 0.1^a^	0.17 ± 0.08^b^	0.19 ± 0.07^b^	0.55 ± 0.2^a^	<.05	NS	NS
Semen calcium (mg/dL)	6.18 ± 1.8^a^	6.6 ± 1.8^a^	6.8 ± 1.6^a^	6.8 ± 1.9^a^	<.05	<.01	<.05

*Note*: a, b, and c indicate the significant differences between the groups at the threshold of 5 or 1%.

### 4.2 Bodyweight and scrotal circumference changes

**FIGURE 1:**
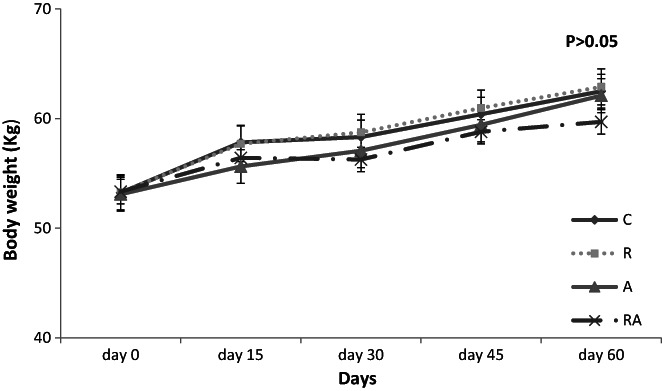
Effect of incorporating *Artemisia herba alba* and *Rosmarinus officinalis* in the diet of rams on bodyweight evolution.

The evolution of the bodyweight of the rams in relation to the diet is presented in Figure 1. The initial weight in the entire group was 53.3 ± 1.2 kg. All groups have shown an increase in live weight during the experiment period as compared to the initial one. At the end of the trial, the highest value was recorded in the R group and the lowest value in the AR group. However, all these differences between groups were not significant (*p* > .05). Only at the end of the experiment, an average difference of 3 kg was recorded between the R rams and the RA rams (*p* < .05).

**FIGURE 2:**
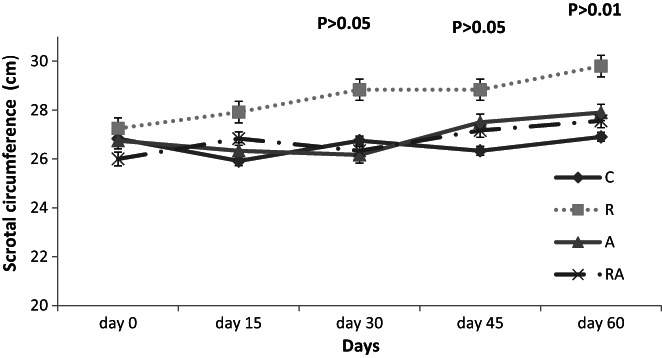
Effect of incorporating *Artemisia herba alba* and *Rosmarinus officinalis* in the diet on the scrotal circumference of rams.

As shown in Figure 2, scrotal circumference increased significantly (*p* < .01) in R rams when compared to C, A and RA rams (28.2 ± 0.40; 26.5 ± 0.40; 26.68 ± 0.40; 26.58 ± 0.40 cm, respectively).

### 4.3 Semen characteristics

All the rams adapted to the semen collection procedures. For experimental rams, changes throughout the experimental phase for the volume of the ejaculate, sperm concentration, and mass activity score are shown in Table 3. Large individual variations marked all the measured semen traits. For pooled rams in the four treatment groups, mean figures were 0.6 ± 0.09 mL for the volume of the ejaculate, 4.83 ± 0.334 billion of spermatozoa/mL for sperm concentration, and 3.35 ± 0.18 for mass activity score.

Sperm volume was not affected by the use of AMP in the diet (*p* > .05) and by the time of sampling (*p* > .5). However, A, R and RA rams consistently had higher sperm concentrations and spermatozoa motility as compared to C animals. Seminal plasma analysis showed that supplementation of rams with Wormwood or Rosemary leaves has a significant effect on calcium concentration (*p* < .05), but no differences for proteins concentration between groups (*p* > .05). The measurement of glucose and seminal insulin showed a decrease in A rams and a decrease in insulin without modification of the glucose concentration in R rams.

### 4.4 Sexual behavior and testosterone level

Sexual behavior tests showed an improvement in the pre‐copulatory behavior studied as well as the libido score in animals supplemented by the leaves of the two aromatic plants (*p* < .05). The improvement was greater in the group that received Artemisia. These behavioral observations were confirmed by the increase in plasma testosterone concentration recorded in these same rams (*p* < .05) (Table 4).

**TABLE 4:** Effect of *Artemisia herba alba* and *Rosmarinus officinalis* leaves supplementation on *ram sexual* behavior and blood testosterone level, means ± SD.

Parameters	Treatment				Effect (*p*)
	C	R	A	RA	Treatment
Lateral approaches	18.3 ± 15.9^a^	22.8 ± 7.8^a^	25.5 ± 15.2^a^	28.3 ± 20.2^a^	NS
Flehmen reaction	3 ± 3.1^a^	3.8 ± 1.5^a^	4.3 ± 1.6^a^	5.8 ± 5.2^a^	NS
Genital sniffing	3.7 ± 3.6^a^	4 ± 2.1^a^	4.5 ± 308^a^	8 ± 5.8^a^	NS
Reaction time (s)	12.3 ± 0.8^a^	1.8 ± 1^b^	1.2 ± 0.4^b^	1 ± 0^b^	<.01
Activity time (s)	406 ± 275^a^	684 ± 120^b^	702 ± 85^b^	815 ± 73^b^	<.01
Mount attempts	2.5 ± 2.4^a^	4.2 ± 2.12^a^	5.8 ± 2.2^a^	6.3 ± 2.8^a^	NS
Score Libido	3.7 ± 2.9^a^	5.5 ± 1.5^b^	7.2 ± 1.3^b^	7.2 ± 1.8^b^	<.05
Testosterone (ng/mL)	5.91 ± 2.47^a^	6.21 ± 2.13^a^	9.54 ± 2.92^b^	9.85 ± 1.95^b^	<.05

*Note*: a, b, and c indicate the significant differences between the groups at the threshold of 5 or 1%.

### 4.5 Blood parameters

The analysis of some blood biochemical markers to study the effect of supplementation of rams with these two aromatic plants showed that the parameters measured remained in the range of the sheep species and had no effect on the general health of the animals. However, a significant decrease in glucose in the RA group and insulin in the R group was recorded. On the other hand, the study of the hepatic function showed an increase in the AST enzyme in RA rams and no effect on the AST enzyme. Plasma cortisol analysis showed a significant increase (*p* < .01) in animals that consume rosemary leaves (R and RA groups) (Table 5).

**TABLE 5:** Effect of *Artemisia herba alba* and of *Rosmarinus officinalis* leaves supplementation on *some* ram blood parameters, means ± SD.

Parameters	Treatment				Effect, *p*		
	C	R	A	RA	Treatment	Time	Treatment × time
Glucose (g/dL)	57.47 ± 5.82^a^	55.76 ± 3.34^a^	59.63 ± 1.76^a^	52.92 ± 2.19^b^	<.01	<.01	NS
Insuline (μg/L)	0.147 ± 0.05^a^	0.100 ± 0.02^a^	0.083 ± 0.02^b^	0.110 ± 0.01^a^	<.01	NS	NS
Protein (g/dL)	7.53 ± 0.13^a^	7.69 ± 0.07^a^	7.3 ± 0.23^a^	7.61 ± 0.23^a^	NS	NS	NS
Urea	15.61 ± 3.44^a^	14.11 ± 2.31^a^	14.34 ± 1.74^a^	15.45 ± 2.28^a^	NS	<.01	NS
NEFA	0.116 ± 0.015^a^	0.105 ± 0.012^a^	0.129 ± 0.012^a^	0.113 ± 0.028^a^	NS	NS	NS
Cortisol (ng/mL)	22 ± 2.03^a^	30.3 ± 3.58^b^	23.85 ± 8.01^a^	27.19 ± 3.41^b^	<.01	<.01	<.05
AST (U/L)	109.52 ± 11.85^a^	97.6 ± 1.42^a^	102 ± 1.01^a^	122 ± 7.57^b^	<.05	NS	NS
ALT (U/L)	24.3 ± 2.04^a^	20.88 ± 1.6^a^	21.53 ± 1.51^a^	24.10 ± 2.59^a^	NS	<.05	NS

Abbreviations: ALT, Alanine aminotransferase; AST, Aspartate aminotransferase; NEFA, Non‐Esterified Fatty Acids.

*Note*: a, b, and c indicate the significant differences between the groups at the threshold of 5 or 1%.

## 5 DISCUSSION

Raising sheep in good environmental and nutritional conditions has always been the concern of breeders throughout the world. This concern is justified by the search for a better quality of meat, sheep milk, and also the reproductive capacity of both males and females and the health of their progenies (Martin & Walkden‐Brown, 1995; Prache & Nozières‐Petit, 2015).

In recent decades, changes have been observed in the procedure of breeding throughout the world and in Tunisia. Indeed, a majority of breeders opt most often nowadays for a sedentary mode and the flocks of sheep are fed either freely in local natural pastures, or with a commercial feed or by combining these two types (Retaillé, 2003; Sicard, 2012; Yabrir et al., 2015). However, due to climate change, with the scarcity of rainfall and the aridity of land, the diet of sheep is largely impacted, which can lead to physiological and behavioral disturbances affecting, in particular, the reproductive system of sheep and their ability to generate offsprings, which is a loss for both the farmers and consumers (Belkadi, 2019; Belkhiri et al., 2019; Pottier et al., 2007).

In order to improve the diet of their sheep flocks when green pastures are scarce, farmers have recourse to commercial feed supplements (Rekik et al., 2007), which are usually quite expensive and not always easily available.

Different breeds of sheep are raised in different regions and in different climatic areas; humid, sub‐humid, semi‐arid, and arid (Bedhiaf‐Romdhani et al., 2008; Sassi‐Zaidy et al., 2014). As a result, the herbaceous flora is much diversified and it strongly impacts the quality of meat, milk and also the vitality and sexual performances of the sheep. Several species of plants are widespread in various regions in Tunisia and they are known for their nutritional virtues and are even used by local populations in their pharmacopeia (Dhaouadi et al., 2013; Riahi‐Chebbi et al., 2016).

Among these species we found *Artemisia herba alba* and *Rosmarinus officinalis*. Several studies have related these two species to the many benefits on human and animal nutrition and health due to their bioactive compounds which have antioxidant, anti‐inflammatory, antimicrobial, anti‐parasitic, anti‐cancer, and anti‐diabetic potentials (Ait‐Kaki et al., 2018; Khlifi et al., 2013; Mohammed et al., 2021; Veenstra & Johnson, 2021).

It has been reported in several studies that the incorporation of plants species in sheep's diet can have an impact on different growth parameters, both muscular and skeletal, as well as on energy metabolisms, certain endocrine pathways, but also on the reproductive function and sexual performance of rams (Aouadi et al., 2014; Baazaoui et al., 2017; Hanafy et al., 2009; Odhaib et al., 2018; Yagoubi et al., 2022).

Considering results from our study, rams diet supplementation with *Artemisia herba alba* or *Rosmarinus officinalis* did not lead to significant changes in blood metabolites, energy and protein parameters since total DMI and chemical composition of diets were similar. Overall, the results of glucose, insulin, NEFA urea, and protein are consistent with dry matter intake (DMI) in all groups. The lowest value of glucose detected in the RA group was in line with the lowest level detected on BW in this group and could be due to the interference of the plant's secondary metabolites on the rumen microbial activity. Actually, rosemary leaves decreased the abundance of archaea, the genus *Prevotella*, *Ruminococcus albus* and *Clostridium aminophilum* of ram rumen (Cobellis et al., 2016) thanks to the antimicrobial property of its active molecules like α‐pinene, β‐pinene, camphene, 1,8 cineole, camphor, borneol, bornyl acetate, and verbenone (Zaouali et al., 2010). Moreover, it has been shown that the main constituents of *Artemisia herba alba* namely cis‐ and trans‐thujone, 1,8‐cineole, camphor, ⍺‐thujone, β‐thujone, and vanillyl alcohol have been reported to have notable antimicrobial activities (Amor et al., 2019; Mighri et al., 2010). On the other hand, the higher level of AST (biomarker of liver activity) found in the RA group could be due to the synergic effects of bioactive metabolites found in the leaves of both *Artemisia herba‐alba* and *Rosmarinus officinalis*, as reported in (Amor et al., 2019; Cobellis et al., 2016; Mighri et al., 2010; Mohamed et al., 2010; Zaouali et al., 2010). The phenolic compounds in rosemary are carnosol, carnosic acid, rosmanol, epirosmanol, isorosmanol, methyl carnosate, and rosmarinic acid known for their antioxidant activities (Nieto et al., 2018). On the other side, *Artemisia herba alba* has antioxidant properties due to its compounds such as sesquiterpene lactones, flavonoids, some phenolic compounds, and waxes which have been found in the plant (Mohamed et al., 2010).

Our results have shown an increase of plasma cortisol level in the supplemented rams. This could be due to the increase of their sexual activity. Indeed, some studies have shown that cortisol levels can be significantly affected by certain sexual practices as it has been reported that sex between men increases plasma corticosterone levels (Borg et al., 1992). Cortisol levels were highest during mating, mounting, and intromission (Borg et al., 1991).

Originally, it was thought that cortisol secretion was mainly due to a reaction to a stressful situation (fear, physical or psychological aggression, climatic aggression, microbial infections, etc.) in order to allow the organism to stay awake and to adapt metabolically to an increased need for energy (Selye, 1956). Over the decades, several studies have shown that cortisol can be secreted by the adrenal glands even in situations of well‐being (intense sports practices, positive emotions in love, etc.). Other studies have reported an increase in plasma corticosterone levels after mating in many mammal species such as stallions, pigs and bulls (Borg et al., 1991; Rabb et al., 1989; Selye, 1956), and rats and mice (Belkadi, 2019; Pottier et al., 2007; Retana‐Marquez et al., 1998), respectively. In (Bonilla‐Jaime et al., 2006), authors have shown that corticosterone levels may enhance up to two‐fold after male exposure to physical or no physical contact with females either in estrous phase or in non‐receptive phase.

Furthermore, it has been shown that during sexual attraction in human courtship, the hypothalamic–pituitary–adrenal axis is stimulated in conjunction with the hypothalamic–pituitary–testicular axis in men when they see a woman with an attractive appearance (van der Meij et al., 2010). This could be explained by the probable existence of active nervous connections between different hypothalamic nuclei.

The incorporation of plants rich in active molecules, such as *Artemisia herba alba* and/or *Rosmarinus officinalis* in the diet of rams, affects their physiology parameters including reproductive activity. The addition of a small quantity of these plants to the daily diet of rams showed an improvement in some reproductive parameters including scrotal circumference, sperm concentration, testosterone level, and sexual behavior in addition to some biochemical changes in blood and sperm. There are few reports on how *Artemisia herba alba* and/or *Rosmarinus officinalis* affects reproduction function (15, 18, 62) many of which targeted in vitro study (Ahmed et al., 2022; Malo et al., 2011; Motlagh et al., 2014) or female reproduction (Akpa et al., 2012; Smeti et al., 2015). To our knowledge, this study is the first to investigate the effects of *Artemisia herba alba* and/or *Rosmarinus officinalis* on rams' reproductive capacity. On the basis of the study findings, some interesting features are being discussed below.

Body performance presents an important parameter for judging the reproductive capacity of a ram (Akpa et al., 2012). In the present study, it has been emphasized that at the end of the experiment we did not record differences in food intake and live weight of all rams increased. However, scrotal circumference was significantly higher in R rams when compared to controls and other experimental groups. In this context, (Khataibeh & Daradka, 2007) showed that intra‐gastric administration of *Artemisia herba alba* caused an increase in BW and in the weight of the testes, epididymides, seminal vesicle, ventral prostate, and vas deferens. However, the ingestion of *Rosmarinus officinalis* did not affect the BW and testes weight, but decreased the weights of epididymides, ventral prostates, seminal vesicles, and preputial glands (Nusier et al., 2007).

Sperm quality is the main factor that limit male reproductive efficiency (Osadchuk & Osadchuk, 2020). Semen traits were affected by the imposed treatment. Indeed, A rams had higher sperm concentrations than C, R and AR animals. However, when rams received *Rosmarinus officinalis* plus *Artemisia herba alba*, the sperm volume increased (*p* > .05). Sperm motility was affected by *Artemisia herba alba*. Contrary to our results, (Khataibeh & Daradka, 2007) showed in males rats an adverse effect of *Artemisia herba alba* on reproductive performance, which was manifested by a decrease in motility and sperm concentration as well as atrophy of the epithelial cells, the seminal vesicle and germ cells including spermatocytes and spermatids.

Seminal plasma supports the physiological phenomena of mammalian gametes (Desnoyers & Manjunath, 1992; Manjunath & Thérien, 2002; Muiño‐Blanco et al., 2008). It is a biological fluid composed of multiple secretions from the testicles, epididymis, prostate, and seminal vesicles (Carvajal‐Serna et al., 2018). Indeed, the study of some biochemical parameters of seminal plasma showed an absence of effect on seminal protein concentration, which presents an important component for the vitality and the fertility of spermatozoa (Carvajal‐Serna et al., 2018; Leahy et al., 2019). Sperm calcium, which plays an important role in sperm motility (Cuevas et al., 2013) was affected by *Rosmarinus officinalis* and *Artemisia herba alba*, which partly explains the plant effect on sperm motility. In contrast to sperm protein, we showed an effect of treatment on the energy metabolism of sperm. In fact, we recorded a decrease in sperm glucose and insulin in A rams because of the positive correlation between them (Elsamanoudy et al., 2016). As shown in the literature, the sugar composition of seminal plasma has been correlated with fertility, mainly because of its importance for sperm energy production (Garner, 2001), and glucose is a source of energy for spermatozoa (Leahy et al., 2019). However, in the present study, the dramatic drop of sperm glucose concentration in rams that received *Artemisia herba alba* separately did not affect the sperm quality parameters. On the contrary, rams that have presented a low level of sperm glucose had the highest concentration of spermatozoa. It has been reported that certain low‐glucose conditions could induce high‐speed linear motility of spermatozoa by activating the mitochondrial activity for ATP generation (Zhu et al., 2019). This suggests a benefit of low glucose concentration on sperm quality. Unfortunately, there are no published results to which these findings could be compared. Moreover, high insulin concentration in seminal fluid caused by obesity reduced male performance (Leisegang et al., 2014). Our results indicate a decrease in insulin level which explains the decrease of glucose thanks to a positive correlation between them (Elsamanoudy et al., 2016). These results may indicate an enhancement of sperm fertility.

Blood testosterone level is an important indicator of fertility and reproductive activity (Apfelbeck et al., 2016; Preston et al., 2012). This steroid hormone promotes sexual behavior and cognitions in order to facilitate mate acquisition or mating directly (Roney et al., 2003). Testosterone levels increase after male sexual activity (Dabbs & Mohammed, 1992).

Indeed, the increase in this parameter shows an increase in the reproductive activity of the animals (Apfelbeck et al., 2016; Preston et al., 2012). In our study, testosterone concentration doubled in rams that received *Artemisia herba alba* in the diet (*p* < .05), which shows an improvement in the reproductive activity of rams. This result is confirmed by sexual behavior tests which showed an improvement in most of the pre‐copulatory behavior (*p* < .01) and libido score (*p* < .05) in rams that received *Artemisia herba alba*. Contrary to our study (Khataibeh & Daradka, 2007) showed a negative effect of *Artemisia herba alba* on the hormonal secretion of follicle‐stimulating hormone (FSH) and blood testosterone in rats.

## 6 CONCLUSION

It emerges from our present study that dietary supplementation of rams with *Artemisia herba alba* or with *Rosmarinus officinalis* has positive effects on their reproductive activity shown by the enhancement of their libido score, testicular circumference, sexual behavior, and fertility as a consequence of the improvement of the spermatic quality as well as the hormonal status regulating the testicular function. This diet could represent a beneficial nutritional potential for the improvement of breeding conditions and development of sheep breeds in Tunisia.

## CONFLICT OF INTEREST STATEMENT

The authors declare that they have no competing interests.

## ETHICS STATEMENT

The authors confirm that the ethical policies of the journal, as noted on the journal's author guidelines page, have been adhered to and the appropriate ethical review committee approval has been received. The authors confirm that they have followed EU standards for the protection of animals used for scientific purposes and feed legislation, if appropriate.

## Data Availability

The data that support the findings of this study are available from the corresponding author upon reasonable request.
